# Use of virtual reality to remotely train healthcare professionals in paediatric emergency tracheostomy skills: protocol for a multi-centre, non-inferiority educational interventional study with historical controls

**DOI:** 10.1186/s12893-024-02736-1

**Published:** 2025-01-15

**Authors:** Jonathan R. Abbas, Noorulanne Younis, Emily Johnstone, Azita Rajai, Rachel Isba, Antony Payton, Brendan A. McGrath, Neil Tolley, Iain A. Bruce

**Affiliations:** 1 Division of Immunology, Immunity to Infection, and Respiratory Medicine, School of Biological Sciences, Faculty of Biology, Medicine and Health, The University of Manchester, Oxford Road, Manchester, M13 9PL UK; 2 Department of Paediatric ENT, Royal Manchester Children’s Hospital, Manchester University Foundation Trust, Oxford Road, Manchester, M13 9WL UK; 3 Research and Innovation, Manchester University NHS Foundation Trust, Manchester Academic Health Science Centre, Hathersage Road, Manchester, M13 9WL UK; 4 Centre for Biostatistics, Division of Population Health, University of Manchester, Manchester Academic Health Science Centre, Manchester, UK; 5 Lancaster Medical School, Lancaster University, Lancaster, LA1 4YW UK; 6 Alder Hey Children’s Hospital NHS Foundation Trust, Liverpool, UK; 7 VREvo Ltd, Umi3 CTF, 46 Grafton Street, Manchester, M13 9NT UK; 8 Manchester Academic Critical Care, Division of Infection, Immunity and Respiratory Medicine, School of Biological Sciences, Faculty of Biology, Medicine and Health,, The University of Manchester, Academic Health Science Centre, Manchester, UK; 9 Department of Anaesthesia, Manchester University NHS Foundation Trust, Manchester, UK; 10 Imperial College NHS Healthcare Trust, St Mary’s Hospital, The Bays, South Wharf Road, London, W2 1NY UK

**Keywords:** Virtual reality, Simulation training, Tracheostomy, Paediatrics

## Abstract

**Background:**

The insertion of a tracheostomy is an established technique used to wean patients off ventilatory support, manage secretions in complex conditions, and as a potentially life-saving procedure to bypass upper airway obstruction. Life-threatening complications during aftercare are not uncommon and may be influenced by a lack of education of carers or healthcare providers of children and young people living with a tracheostomy. Education programmes designed and supported by the National Tracheostomy Safety Project are effective, but resources are not available to educate the workforce at scale. With the overarching aim of widening access to paediatric tracheostomy skills training, we present the protocol for the development and evaluation of a novel virtual reality (VR) training tool designed to simulate the emergency management of paediatric tracheostomy complications.

**Methods and discussion:**

A multi-centre, non-inferiority educational interventional study with historical controls will be used to evaluate the novel VR training package. A group of 69 healthcare staff and students will have one week to use the educational intervention as often as necessary to learn paediatric emergency tracheostomy skills. The primary outcome measure is skill performance in simulation in a pre- and post-intervention structure within subjects. Participant performance will also be assessed using non-inferiority metrics against historical traditional educational control data. Secondary outcomes include knowledge gain, knowledge retention, usability, side effects, and participant satisfaction. To minimise the risk of cybersickness, teleportation was the preferred locomotion method for the user navigation within the VR environment.

**Trial registration:**

Full registration of this study was completed at ClinicalTrials.gov. The registration number is NCT06350708 and was accepted on the 4th April 2024.

**Supplementary Information:**

The online version contains supplementary material available at 10.1186/s12893-024-02736-1.

## Introduction

The emergency management of the paediatric tracheostomy represents a critical intervention for children and young people (CYP) living with a tracheostomy [1]. Comprehensive training is indispensable to ensure this vulnerable group's safe and effective care. However, traditional tracheostomy education currently available online, involves limited access to practical training opportunities. Therefore, the healthcare workforce, both in the community and in secondary care, relies heavily on in-person teaching methods [2]. This approach poses logistical challenges and can carry cost and environmental impact considerations due to trainer and trainee travel, equipment, and disposable single-use use consumables [3].

In recent years, virtual reality (VR) technology has emerged as a promising tool for healthcare training, offering potentially immersive and realistic simulations that can replicate sometimes complex emergency scenarios [4–6]. In this protocol, we present the planned testing of a state-of-the-art paediatric VR tracheostomy training tool designed to provide students with limitlessly repayable education while enabling remote assessment of their knowledge acquisition.

The primary aim of this study is to examine the effectiveness of the VR paediatric tracheostomy training tool in facilitating learning and enhancing students’ understanding of the emergency management protocol as described by the National Tracheostomy Safety Project (NTSP) (www.tracheostomy.org) [7]. The NTSP blocked tracheostomy protocol is the current 'gold standard' management algorithm taught in UK hospitals using traditional mannequin-based simulation [7]. By providing a safe and controlled virtual environment, we aim to enable learners to engage in hands-on practice, hone their practical emergency algorithm skills, and gain confidence in caring for CYP living with a tracheostomy. Academics and institutions have been exploring VR as a medical education tool over the past two decades [8]. Despite the dramatic increase in consumer use, implementation in the UK healthcare system is limited, with several contributing factors including lack of understanding, confusion in the literature around terminology, the likelihood of cybersickness and no standard way of distributing education using these technologies [8]. Several options exist, including using VR as part of medical courses, using VR on-site in physical settings such as hospitals, universities, or another educational setting, or fully-remote deployments that allow learners to engage with VR content from home or location via online platforms [9]. Additionally, the NTSP have developed a VR course to train in adult tracheostomy safety skills in a supervised remote model [10]. Distance learning has the advantage of liberating trainers and not requiring specialised spaces within healthcare settings. However, the learner cannot utilise the value of real-time educator-student interaction. This article contributes to the growing body of research on distance based VR education in healthcare. By testing the effectiveness of a novel paediatric VR tracheostomy training tool, we aim to explore the acceptability and educational equivalence of VR versus traditional simulation training for the acquisition of knowledge and skills required to manage the paediatric tracheostomy emergency.

### Overall aim and research questions

The novel VR tracheostomy training package will be used as described in Table 1. By evaluating this novel VR training package, we aim to explore whether healthcare students can gain knowledge and skills relevant to managing paediatric tracheostomy emergencies using immersive technology in their homes. In addition to skill acquisition and knowledge, we aim to understand the usability of these devices in the remote setting and learn about the user's satisfaction levels and perception of realism by utilising the training package in a self-directed manner. The research questions are as follows:

1. Is fully remote, unsupervised VR training non-inferior to traditional training?
2. Do participants enjoy and feel they can learn from fully remote VR training?

**Table 1:** Summary of the VR educational activity

Activity	Detail
**Onboarding (5 min)**	This 5-min experience guides the participant through using the controllers to interact with the environment and virtual objects and move around. It teaches the user how to perform specific interactions, such as suctioning the tracheostomy and speaking to the avatar in the simulation
**History taking (5–10 min)**	The user is expected to take a brief history from a simulated avatar of the patient’s mother as they have attended the emergency department due to a cough and fever. The user will be able to ask relevant questions verbally and be responded to by the avatar. The aim is to gather any information regarding when the tracheostomy was inserted, when the tracheostomy was last suctioned, any allergies the patient may have, and whether the patient has eaten before coming to the hospital. This is to determine whether the procedure can go forth
**Algorithm tutorial training (5–10 min)**	This is a guided tutorial that demonstrates and allows the user to practice using the emergency tracheostomy algorithm. It introduces the blue emergency box, algorithm card, and equipment required to complete the process of decannulating the child. There are no elements of stress in this scene; it is purely a learning space
**Algorithm simulation assessment (5–10 min)**	This section will expose the user to an emergency and require them to progress through the blocked tracheostomy algorithm Feedback is given to the user around time, including interventions they have completed and ones they missed The user can repeat the assessment or the tutorial to improve their time

## Materials and methods

The planned study is a multi-centre non-inferiority educational interventional study with historical controls. Historical control participants undertook a simulation course designed to train individuals in caring for the paediatric tracheostomy patient, which was run by the Paediatric Working Group of the NTSP (www.tracheostomy.org). This data set was published as a scientific abstract in 2015 in the British Journal of Anaesthesia and was available to the study management group [11]. The educational intervention will provide access to a remote, unsupervised VR training package designed to teach the management of the paediatric tracheostomy emergency using a nationally established emergency algorithm.

The VR training package has four key sections in which the user must complete:

1. Onboarding Section: This section provides instruction on how to navigate the virtual environment familiarising the user with the controls and interactions used within the simulation.
2. History-Taking Section: Prior to proceeding with the main simulation**.** Users engage in a verbal conversation with an AI avatar to ask relevant questions and gather relevant information regarding the tracheostomy.
3. Algorithm Tutorial Section:
4. This section is a step-by-step guide aligned with the national algorithm card. Each step and interaction are explained and highlighted in the correct sequence, allowing the user to acquaint themselves with the process.
5. Algorithm Simulation Assessment: This section exposes the user to an emergency scenario with added triggers to simulate high pressure conditions. The user is expected to perform the correct sequence of steps without assistive prompts within a certain timeframe, this allows them to practice critical decision-making. Once they have completed the assessment, detailed feedback is provided including the number and sequence of steps performed along with the time taken. Users can identify areas of improvement and repeat the algorithm tutorial and assessment section until they feel competent for real-life scenarios.

To minimise the risk of cybersickness, teleportation was the preferred locomotion method for the user navigation within the VR environment [12]. Traditional VR movement options such as joystick-based locomotion can cause discomfort and disorientation due to mismatched sensory feedback. Teleportation on the other hand, allows the user to snap from one point to another reducing the likelihood of nausea and dizziness. Additionally, teleportation contributed to the performance of the simulation by keeping the frames per second (FPS) at a steady 72, which is a crucial factor when creating smooth and responsive experiences. Higher FPS ensures the virtual environment, interactive objects and animations are rendered smoothly, reducing lag that could negatively impact user experience and immersion.

### Participants

#### Recruitment and consent of participants

This study will recruit healthcare staff and students from the University of Manchester (UoM) and Manchester University NHS Foundation Trust (MFT). Recruitment into this study will be advertised during routine lectures, posters, ‘word of mouth’, snowball recruitment (asking participants to recommend further people to participate), UoM and MFT Bulletins, social media feeds, and noticeboards [13]. Advertisements contain a quick response (QR) code directing the individual to a participant information sheet (PIS) and consent form. On completion, they will be individually contacted, and instructions will be given on how to proceed with the study. Consent will be confirmed upon attendance at the VR simulation laboratory in UoM or a simulation skills area in MFT. Withdrawal from enrolment into this study is possible at any time, without stating any reason, and without any detriment to the participant.

#### Inclusion and exclusion criteria

For this study, specific inclusion and exclusion criteria have been defined ahead of the recruitment start date, and documented in the ethics application. These are listed below:

- Inclusion criteria are as follows:
  - Healthcare student
  - Clinical experience needed
  - Healthcare staff
  - Based at MFT, or The UoM
- Exclusion criteria are as follows:
  - Not a healthcare student
  - Not a healthcare student—have no clinical experience
  - Not healthcare staff but working in the NHS
  - Not based at MFT, or The UoM

### Description of the intervention

The educational tool is a stand-alone software package hosted entirely on a VR HMD. It has been developed by VREvo Ltd www.sentiraxr.com. It is designed to be used without direct supervision by a technician or educator and to teach the following learning outcomes, model of the long-running NTSP paediatric tracheostomy safety course:

1. Recognise that paediatric tracheostomy emergencies require placement of oxygenation via the mouth and nose.
2. Recall critical equipment required during a blocked tracheostomy emergency.
3. Learn to follow the NTSP paediatric emergency tracheostomy algorithm and, in doing so, practice the following steps to solve the emergency:
4. Approaching the child should include safety, stimulation, shouting for help, and oxygen.
5. Give oxygen via an appropriate route.
6. Remove external attachments from the tracheostomy tube, e.g., the speaking valve.
7. Using a suction catheter, Determine if the tracheostomy is patent or blocked.
8. Perform an emergency tube change and reassess patency after the change.
9. If the emergency tube change does not restore the airway, give five rescue breaths at an appropriate point in the resuscitation, using a suitable device and entry point (mouth or stoma).
10. Experience working through the algorithm with various external and internal pressures and stressors to mimic real-world use.

The software package takes around 20–35 min to complete and has four distinct activities within the virtual environment. These are detailed in Table 1:

Following recruitment and electronic consent process, the participants will attend the study site (MFT or The UoM). At this point, following confirmation of consent, demographic data and the pre-course knowledge test will be administered (Appendix 1). Following this, the individual will be required to manage a standardised simulated blocked paediatric tracheostomy, which will be recorded, and performance data will be logged. The participants will then be provided with the VR training loaded onto a Meta Quest 3 head-mounted display (HMD) which they will take for one week. The participants will be expected to use the application at least four times, but up to as many times as required until they feel they have learnt the content effectively. They will be required to record their use, and we will record how many times it has been used and for how long (Appendix 1).

**Fig. 1:**
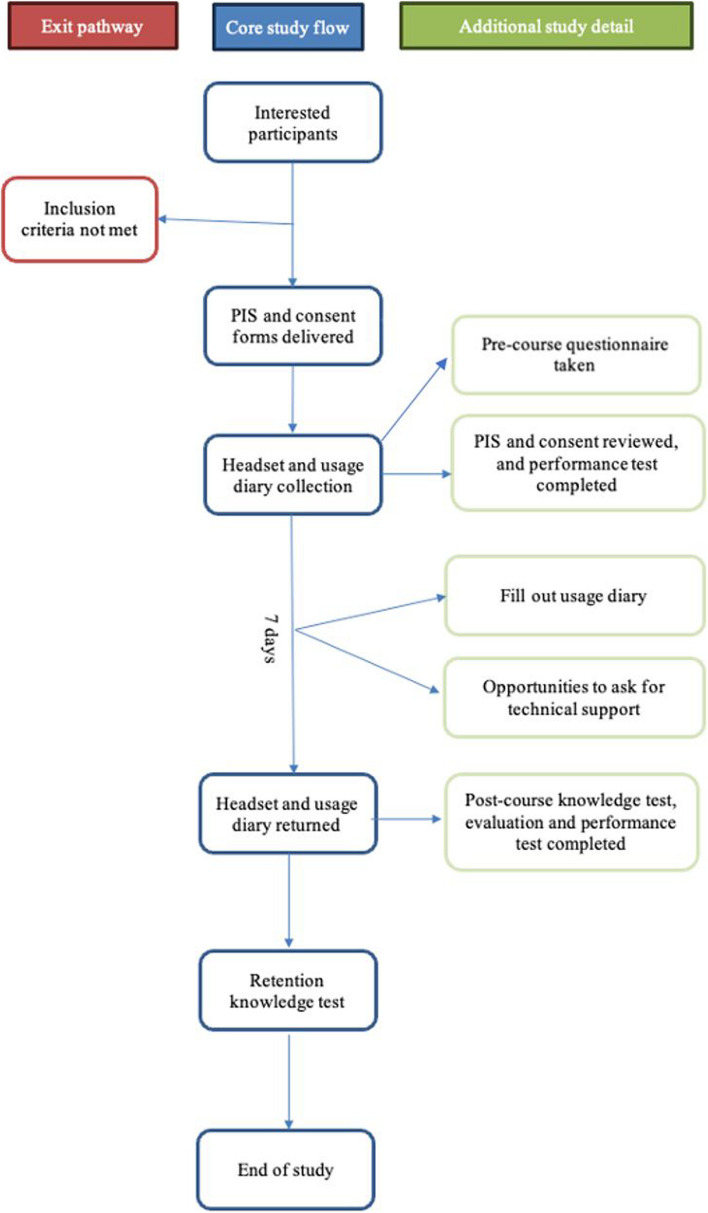
Study flow diagram demonstrating the process from recruitment to study completion

During the study, the participants will have access to an email address to ask technical support questions and open virtual calls regularly during the week if they need verbal technical support. The participant will be able to get in-person technical support at the university if required. All technical support requests will be documented and reported (Appendix 1). At the end of the one-week study period, the participant must return the HMD to the VR laboratory or simulation area and undertake a second simulated assessment. The outcome measures in these simulated assessments are identical to those completed in the study from which we will be taking historical controls and will form the basis for statistical comparisons. This scenario will teach and assess the same principles as the first but differ in specific clinical situations. It will be followed by a de-brief for educational purposes, which will not form part of the assessment or be recorded. The participant will also complete the electronic post-course questionnaire with a repeat knowledge test (Appendix 1). Finally, an electronic knowledge retention test, identical to the previous knowledge tests, will be completed four weeks after the post-course questionnaire. A certificate of participation, including a £75.00 incentive voucher, will be delivered electronically upon completion of the retention test. The study flow is diagrammatically represented in Fig. 1.

### Statistical calculations

Sample size calculations were performed by an MFT statistician (AR) to assess for non-inferiority of VR training compared to the current standard training. In the historical data set, the standard deviation for total scenario time after standard training was 70 s. The non-inferiority margin is assumed to be 30 s. Assuming the expected time to key specific interventions and completion of the scenario for the two groups are the same, the sample size is calculated so that the upper limit of the one-sided confidence interval for the mean difference in time (VR- standard) is below 30 s. A 97.5% one-sided confidence interval for non-inferiority translates to a 95% two-sided confidence interval, provided the upper limit is below 30 s. The calculation provided led to a proposed sample size of 69 participants. Descriptive statistics will be obtained from the pre and post course surveys and the assessment data. Secondly aggregate measures will be constructed for further analysis. Examples of aggregate measures include:

1. Mean values for the simulation performance (primary outcome)
2. Mean values for knowledge test score

The data will be compared to historical data from a course designed to teach the same learning outcomes. Specific measures for performance will be compared, including total scenario completion time and time to specific key interventions.

### Outcome measures

The process for assessing primary and secondary outcomes throughout the study period are outlined below. All of the data collection points are included in Appendix 1.

### Primary outcome—assessment of performance in simulation

The participants synthesised knowledge will be assessed before and after the intervention during a traditional simulation skills session. Both sessions will include a pre-brief, with the second session following up with a de-brief pedagogical methodology as per best standards in simulation design [14]. The simulation will have the same layout and equipment as the VR training tool. These sessions will be analysed on-site, and the following data will be taken:

1. Overall success in task (measured as a binary ‘yes’ or ‘no’ outcome)
2. Steps completed (all measured as a binary ‘yes’ or ‘no’ for each of the below individual steps marked a-g)
  - Call for help
  - Look / listen / feel
  - Apply oxygen – face
  - Apply oxygen – tracheostomy
  - Remove any attachments
  - Attempt suction
  - Remove tracheostomy
3. Time to key interventions (measured in seconds for all individual steps below marked a-c)
  - Apply oxygen to face and neck
  - Call for help
  - Completion of simulation

### Secondary Outcomes

1. Knowledge – multiple-choice question (MCQ) knowledge test – validated 10-question MCQ test, marked out of a total score of 23. This knowledge test will be based on the learning outcomes of the course and adapted from the National Tracheostomy Safety Project (NTSP) – E-Leaning for Healthcare modules [15]. These are well-established knowledge questions with electronic modules that thousands of participants have taken. The knowledge test will not be ‘validated’ for research purposes; however, it is certainly valid regarding face and content validity. The knowledge test will be administered four weeks following the intervention to measure retention.
2. Participant satisfaction – Likert style questionnaire – Satisfaction questionnaire evaluating the use of VR for this education type and questions around the feasibility of doing this at home, unsupervised. Questions include a five-point Likert scale, true and false answers, and white space questions.
3. Comfort and side effects – validated Virtual Reality Sickness Questionnaire [16]**—**this validated questionnaire assesses all possible VR sickness symptoms and will be delivered post intervention. The format of this questionnaire is a four-point Likert scale.
4. Usability – validated System Usability Scale [17]**—**widely accepted questionnaire used to assess technology usability. The format of this questionnaire is a five-point Likert scale.

### Patient and Public Involvement (PPI)

Ahead of the study design and novel VR tool development, PPI activity supported by the National Institute for Healthcare Research (NIHR) Research Design Service PPI fund was undertaken. Using structured interviews, the research team discussed the project with individuals working or studying in medical or paramedical healthcare roles. This process actively impacted the design of the educational intervention and study.

### Compliance with ethical standards

This study has been assessed and approved by The UoM Research Ethics Committee (2023–18304-31443) and Health Research Authority (336664), and adoption into the National Institute for Health and Care Research: Clinical Research Network Portfolio is complete. The study was managed according to the European General Data Protection Regulation and the internationally accepted Good Clinical Practice Guidelines [18–20]. Additionally, this study has been registered on the clinical trials registry ClinicalTrials.gov [21]. All participants receive a PIS at least 48 h prior to the study commencement. Given that VR is generally well tolerated, adverse effects were not expected, but despite this, a full general distress protocol was created [22, 23]. It was made clear that participating in this study was voluntary, and withdrawal from the study was possible without providing reason or any consequence at any time.

## Supplementary Information

Supplementary Material 1.

Supplementary Material 2.

Supplementary Material 3.

Supplementary Material 4.

Supplementary Material 5.

Supplementary Material 6.

## Data Availability

No datasets were generated or analysed during the current study.
