# Supplementary material for: Use of virtual reality to remotely train healthcare professionals in paediatric emergency tracheostomy skills: protocol for a multi-centre, non-inferiority educational interventional study with historical controls
Source: BMC Surg. 2025 Jan 15;25:25. doi: 10.1186/s12893-024-02736-1 (PMC11734328; doi:10.1186/s12893-024-02736-1)
Supplement: Supplementary file 2 — Supplementary Material 2. [file 12893_2024_2736_MOESM2_ESM.docx]

**VR activity diary.**

Use the diary below to indicate when you have used the software, and how many times you used it in each session. An example filled diary is on page 2.

|  | **Day 1** | **Day 2** | **Day 3** | **Day 4** | **Day 5** | **Day 6** | **Day 7** |
| --- | --- | --- | --- | --- | --- | --- | --- |
| **Morning**  **05:00-12:00** |  |  |  |  |  |  |  |
| **Afternoon**  **12:00 – 17:00** |  |  |  |  |  |  |  |
| **Evening**  **17:00-22:00** |  |  |  |  |  |  |  |
| **Night**  **22:00-05:00** |  |  |  |  |  |  |  |
|  | **Day 1** | **Day 2** | **Day 3** | **Day 4** | **Day 5** | **Day 6** | **Day 7** |
| **Morning**  **05:00-12:00** | **1** |  | **1** |  |  |  | **1** |
| **Afternoon**  **12:00 – 17:00** |  | **2** |  |  |  | **1** | **1** |
| **Evening**  **17:00-22:00** |  |  |  | 3 |  |  |  |
| **Night**  **22:00-05:00** |  | **1** |  |  |  |  | 3 |
