# Supplementary material for: Use of virtual reality to remotely train healthcare professionals in paediatric emergency tracheostomy skills: protocol for a multi-centre, non-inferiority educational interventional study with historical controls
Source: BMC Surg. 2025 Jan 15;25:25. doi: 10.1186/s12893-024-02736-1 (PMC11734328; doi:10.1186/s12893-024-02736-1)
Supplement: Supplementary file 3 — Supplementary Material 3. [file 12893_2024_2736_MOESM3_ESM.docx]

**Technical support request record**

| **Participant ID** | **Support date / time** | **Severity** | **Detail** | **Solution** |
| --- | --- | --- | --- | --- |
|  |  |  |  |  |
|  |  |  |  |  |
|  |  |  |  |  |
|  |  |  |  |  |
