# Supplementary material for: Use of virtual reality to remotely train healthcare professionals in paediatric emergency tracheostomy skills: protocol for a multi-centre, non-inferiority educational interventional study with historical controls
Source: BMC Surg. 2025 Jan 15;25:25. doi: 10.1186/s12893-024-02736-1 (PMC11734328; doi:10.1186/s12893-024-02736-1)
Supplement: Supplementary file 5 — Supplementary Material 5. [file 12893_2024_2736_MOESM5_ESM.docx]

**Retention questionnaire**

Enter your participant ID:

**Knowledge Questionnaire**

1. State whether this statement is true or false. In a paediatric tracheostomy emergency, oxygen should be applied to the mouth AND neck.
   1. True
   2. False
2. Which of the following are recognised indications for a temporary tracheostomy? Select all that are correct.
   1. Inability to protect the airway after a head injury
   2. Severe facial trauma
   3. Surgical removal of carcinoma of the larynx
   4. Weaning from mechanical ventilation
   5. Inability to clear secretions
3. In a tracheostomy emergency which of the following accessories should be removed prior to performing suction. Select all that are correct.
   1. Heat Moisture Exchange
   2. Speaking valve
   3. Oxygen Facemask
   4. Inner tube
   5. Tracheostomy dressing
4. Which of the following statements are appropriate initial interventions for a deteriorating child who is NOT breathing, following removal of a blocked tracheostomy. Select all that are correct.
5. Apply chest compressions
6. Intubate the stoma with a 6.0 mm endotracheal tube
7. Intubate the stoma with a new 7.0 cuffed tracheostomy tube
8. Give rescue breaths to the tracheostomy stoma
9. Give rescue breaths orally
10. The following situations could be considered tracheostomy 'Red Flags'. Select all that are correct.
    1. Suction catheter only passes intermittently
    2. Patient has an uncuffed tube in situ, breathing spontaneously via trachy-mask, and can talk softly
    3. There is no cuff on the tube
    4. The child has low oxygen saturations
    5. The child is blue
11. Which of the following statements is not true about caring for a child with a tracheostomy tube in situ? Select all that are incorrect.
    1. A child may have a cuffed or an uncuffed tube
    2. You cannot give rescue breaths orally
    3. Basic life support principles apply
    4. Advanced paediatric life support principles apply
    5. A child with a tracheostomy may be able to vocalise
12. In the emergency algorithm for a child with a tracheostomy emergency, the next step after being unable to pass a suction catheter down the tube is to remove the tracheostomy and replace it with a smaller tube.
    1. True
    2. False
13. If replacement of the tube has been replaced with the same size, and then a smaller size the next step in the emergency algorithm is to remove it altogether.
    1. True
    2. False
14. If a child has had their tube removed during a tracheostomy emergency, it is unsafe to try and ventilate them using a bag, valve, mask over the stoma in the neck.
    1. True
    2. False
15. Which of the following methods may be useful when assessing for signs of breathing during a tracheostomy emergency? Select all that are correct.
16. Look at the rise and fall of the chest
17. Listen for breath sounds at the neck
18. Listen for breath sounds at the mouth
19. Fell for breath sounds at the neck
20. Feel for breath sounds at the mouth
