# Supplementary material for: Use of virtual reality to remotely train healthcare professionals in paediatric emergency tracheostomy skills: protocol for a multi-centre, non-inferiority educational interventional study with historical controls
Source: BMC Surg. 2025 Jan 15;25:25. doi: 10.1186/s12893-024-02736-1 (PMC11734328; doi:10.1186/s12893-024-02736-1)
Supplement: Supplementary file 6 — Supplementary Material 6. [file 12893_2024_2736_MOESM6_ESM.pdf]

Pre – course questionnaire

Enter your participant ID:

Demographic Questions

Demographic	Further detail
**Age**	16-21<br>21-25<br>26-30<br>31-35<br>36-40<br>41-45<br>46-50<br>51-55<br>56-60<br>Prefer not to say
**Gender**<br><br>As recommended by the UoM approved survey software - Qualtrics	Which gender identity do you most identify with?<br><br>1. Female<br>2. Male<br>3. Non-binary / third gender<br>4. Other (free-text)<br>5. Prefer not to say
**Ethnicity**<br><br>As per Office of National Statistics (ONS) 2021 census guideline.	- White - Includes British, Northern Irish, Irish, Gypsy, Irish Traveller, Roma or any other White background.<br><br>-Mixed or Multiple ethnic groups<br>Includes White and Black Caribbean, White and Black African, White and Asian<br>or any other Mixed or Multiple background-Asian / Asian British<br><br>- Asian or Asian British<br>(Includes Indian, Pakistani, Bangladeshi, Chinese or any other Asian background).<br><br>- Black, Black British, Caribbean or African<br>Includes Black British, Caribbean, African or any other Black background<br><br>-Other - Includes Arab or any other ethnic group
**Discipline**	Medical<br>Nursing<br>Physiotherapy<br>Speech and Language Therapy<br>Operating Theatre Practitioner<br>Other (free-text)
**Professional level**	Student<br>Foundation doctor

	Middle grade doctor<br>Consultant doctor<br>Band 5<br>Band 6<br>Band 7<br>Band 8+
**Experience with VR**	Nil<br>Novice (VR experience < 3 hrs)<br>Intermediate (VR experience 3-24 hrs)<br>Expert (>24 hrs VR experience)

<u>Knowledge Questionnaire</u>

1. State whether this statement is true or false. In a paediatric tracheostomy emergency, oxygen should be applied to the mouth AND neck.

   a. True
   b. False

2. Which of the following are recognised indications for a temporary tracheostomy? Select all that are correct.

   a. Inability to protect the airway after a head injury
   b. Severe facial trauma
   c. Surgical removal of carcinoma of the larynx
   d. Weaning from mechanical ventilation
   e. Inability to clear secretions

3. In a tracheostomy emergency which of the following accessories should be removed prior to performing suction. Select all that are correct.

   a. Heat Moisture Exchange
   b. Speaking valve
   c. Oxygen Facemask
   d. Inner tube
   e. Tracheostomy dressing

4. Which of the following statements are appropriate initial interventions for a deteriorating child who is NOT breathing, following removal of a blocked tracheostomy. Select all that are correct.

   A- Apply chest compressions
   B- Intubate the stoma with a 6.0 mm endotracheal tube
   C- Intubate the stoma with a new 7.0 cuffed tracheostomy tube
   D- Give rescue breaths to the tracheostomy stoma
   E- Give rescue breaths orally

5. The following situations could be considered tracheostomy 'Red Flags'. Select all that are correct.

   a. Suction catheter only passes intermittently

b. Patient has an uncuffed tube in situ, breathing spontaneously via trachy-mask, and can talk softly
c. There is no cuff on the tube
d. The child has low oxygen saturations
e. The child is blue

6. Which of the following statements is not true about caring for a child with a tracheostomy tube in situ? Select all that are incorrect.

   a. A child may have a cuffed or an uncuffed tube
   b. You cannot give rescue breaths orally
   c. Basic life support principles apply
   d. Advanced paediatric life support principles apply
   e. A child with a tracheostomy may be able to vocalise

7. In the emergency algorithm for a child with a tracheostomy emergency, the next step after being unable to pass a suction catheter down the tube is to remove the tracheostomy and replace it with a smaller tube.

   a. True
   b. False

8. If replacement of the tube has been replaced with the same size, and then a smaller size the next step in the emergency algorithm is to remove it altogether.

   a. True
   b. False

9. If a child has had their tube removed during a tracheostomy emergency, it is unsafe to try and ventilate them using a bag, valve, mask over the stoma in the neck.
   a. True
   b. False

10. Which of the following methods may be useful when assessing for signs of breathing during a tracheostomy emergency? Select all that are correct.

   a. Look at the rise and fall of the chest
   b. Listen for breath sounds at the neck
   c. Listen for breath sounds at the mouth
   d. Fell for breath sounds at the neck
   e. Feel for breath sounds at the mouth

VR activity diary.

Use the diary below to indicate when you have used the software, and how many times you used it in each session. An example filled diary is on page 2.

	Day 1	Day 2	Day 3	Day 4	Day 5	Day 6	Day 7
**Morning 05:00-12:00**							
**Afternoon 12:00 – 17:00**							
**Evening 17:00-22:00**							
**Night 22:00-05:00**							

	Day 1	Day 2	Day 3	Day 4	Day 5	Day 6	Day 7
**Morning 05:00-12:00**	1		1				1
**Afternoon 12:00 – 17:00**		2				1	1
**Evening 17:00-22:00**				3			
**Night 22:00-05:00**		1					3

Technical support request record

Participant ID	Support date / time	Severity	Detail	Solution

Post-Intervention questionnaire

Enter your participant ID:

Knowledge Questionnaire

1. State whether this statement is true or false. In a paediatric tracheostomy emergency, oxygen should be applied to the mouth AND neck.

   a. True
   b. False

2. Which of the following are recognised indications for a temporary tracheostomy? Select all that are correct.

   a. Inability to protect the airway after a head injury
   b. Severe facial trauma
   c. Surgical removal of carcinoma of the larynx
   d. Weaning from mechanical ventilation
   e. Inability to clear secretions

3. In a tracheostomy emergency which of the following accessories should be removed prior to performing suction. Select all that are correct.

   a. Heat Moisture Exchange
   b. Speaking valve
   c. Oxygen Facemask
   d. Inner tube
   e. Tracheostomy dressing

4. Which of the following statements are appropriate initial interventions for a deteriorating child who is NOT breathing, following removal of a blocked tracheostomy. Select all that are correct.

   A- Apply chest compressions
   B- Intubate the stoma with a 6.0 mm endotracheal tube
   C- Intubate the stoma with a new 7.0 cuffed tracheostomy tube
   D- Give rescue breaths to the tracheostomy stoma
   E- Give rescue breaths orally

5. The following situations could be considered tracheostomy 'Red Flags'. Select all that are correct.

   a. Suction catheter only passes intermittently
   b. Patient has an uncuffed tube in situ, breathing spontaneously via trachy-mask, and can talk softly
   c. There is no cuff on the tube
   d. The child has low oxygen saturations
   e. The child is blue

6. Which of the following statements is not true about caring for a child with a tracheostomy tube in situ? Select all that are incorrect.

   a. A child may have a cuffed or an uncuffed tube
   b. You cannot give rescue breaths orally
   c. Basic life support principles apply
   d. Advanced paediatric life support principles apply
   e. A child with a tracheostomy may be able to vocalise

7. In the emergency algorithm for a child with a tracheostomy emergency, the next step after being unable to pass a suction catheter down the tube is to remove the tracheostomy and replace it with a smaller tube.

   a. True
   b. False

8. If replacement of the tube has been replaced with the same size, and then a smaller size the next step in the emergency algorithm is to remove it altogether.

   a. True
   b. False

9. If a child has had their tube removed during a tracheostomy emergency, it is unsafe to try and ventilate them using a bag, valve, mask over the stoma in the neck.
   a. True
   b. False

10. Which of the following methods may be useful when assessing for signs of breathing during a tracheostomy emergency? Select all that are correct.

   a. Look at the rise and fall of the chest
   b. Listen for breath sounds at the neck
   c. Listen for breath sounds at the mouth
   d. Fell for breath sounds at the neck
   e. Feel for breath sounds at the mouth

Participant Satisfaction Survey

1. Overall I found the system useful when learning how to manage a paediatric tracheostomy emergency:

    - Strongly agree
    - Agree
    - Neutral
    - Disagree
    - Strongly disagree

Space for entry of additional comments

2. The onboarding section was useful in orientating me how to use virtual reality:

    - Strongly agree
    - Agree
    - Neutral
    - Disagree
    - Strongly disagree

3. The paediatric tracheostomy tutorial section was useful:

    - Strongly agree
    - Agree
    - Neutral
    - Disagree
    - Strongly disagree

4. I feel that I would recognise the required equipment in an emergency more readily after using the VR training:

    - Strongly agree
    - Agree
    - Neutral
    - Disagree
    - Strongly disagree

5. I feel that I will be less stressed when facing a paediatric tracheostomy emergency after using this VR training:

    - Strongly agree
    - Agree

- Neutral
- Disagree
- Strongly disagree

6. Do you feel this VR training experience has the ability to improve patient safety?

    - Yes
    - No

7. Do you feel this VR training experience has the ability to improve your performance within an emergency team?

    - Yes
    - No

8. Do you feel comfortable using the VR training at home?

    - Yes
    - No

9. Would you feel more comfortable using the VR training in a hospital or at university?

    - Yes
    - No

10. Do you feel VR training is an efficient and effective education modality for healthcare staff?

    - Yes
    - No

11. How do you rate the VR education experience overall?

- 1- very poor
- 2
- 3
- 4
- 5- excellent

12.Please add any additional comments (positive or negative) about the VR system, hardware, software or anything that may be relevant.

Enter your answer

VR Sickness Questionnaire

During your experience of the using the virtual reality training, did you experience any of the following symptoms?

Please score each symptom from 0-3 depending on the severity.
(None = 0, Slight = 1, Moderate = 2, Severe = 3)

- General discomfort

0	1	2	3

- Fatigue

0	1	2	3

- Eye Strain

0	1	2	3

- Difficulty focusing

0	1	2	3

- Headache

0	1	2	3

- Fullness of head

0	1	2	3

- Blurred vision

0	1	2	3

- Dizzy (eyes closed)

0	1	2	3

- Vertigo

0	1	2	3

System Usability Scale

Please score the following 10 statements relating to your experience with the virtual reality headset.

(1= Strongly disagree, 5= Strongly agree)

1. I think that I would like to use this system frequently.

1	2	3	4	5

2. I found the system unnecessarily complex.

1	2	3	4	5

3. I though the system was easy to use.

1	2	3	4	5

4. I think that I would need the support of a technical person to be able to use the system.

1	2	3	4	5

5. I found the various functions in the systems were well integrated.

1	2	3	4	5

6. I thought there was too much inconsistency in the system.

1	2	3	4	5

7. I would imagine that most people would learn to use the system quickly.

1	2	3	4	5

8. I found the system cumbersome to use.

1	2	3	4	5

9. I felt very confident using the system.

1	2	3	4	5

10. I needed to learn a lot before I could get going with the system.

1	2	3	4	5

Retention questionnaire

Enter your participant ID:

<u>Knowledge Questionnaire</u>

1. State whether this statement is true or false. In a paediatric tracheostomy emergency, oxygen should be applied to the mouth AND neck.

   a. True
   b. False

2. Which of the following are recognised indications for a temporary tracheostomy? Select all that are correct.

   a. Inability to protect the airway after a head injury
   b. Severe facial trauma
   c. Surgical removal of carcinoma of the larynx
   d. Weaning from mechanical ventilation
   e. Inability to clear secretions

3. In a tracheostomy emergency which of the following accessories should be removed prior to performing suction. Select all that are correct.

   a. Heat Moisture Exchange
   b. Speaking valve
   c. Oxygen Facemask
   d. Inner tube
   e. Tracheostomy dressing

4. Which of the following statements are appropriate initial interventions for a deteriorating child who is NOT breathing, following removal of a blocked tracheostomy. Select all that are correct.

   A- Apply chest compressions
   B- Intubate the stoma with a 6.0 mm endotracheal tube
   C- Intubate the stoma with a new 7.0 cuffed tracheostomy tube
   D- Give rescue breaths to the tracheostomy stoma
   E- Give rescue breaths orally

5. The following situations could be considered tracheostomy 'Red Flags'. Select all that are correct.

   a. Suction catheter only passes intermittently
   b. Patient has an uncuffed tube in situ, breathing spontaneously via trachy-mask, and can talk softly
   c. There is no cuff on the tube
   d. The child has low oxygen saturations
   e. The child is blue

6. Which of the following statements is not true about caring for a child with a tracheostomy tube in situ? Select all that are incorrect.

   a. A child may have a cuffed or an uncuffed tube
   b. You cannot give rescue breaths orally
   c. Basic life support principles apply
   d. Advanced paediatric life support principles apply
   e. A child with a tracheostomy may be able to vocalise

7. In the emergency algorithm for a child with a tracheostomy emergency, the next step after being unable to pass a suction catheter down the tube is to remove the tracheostomy and replace it with a smaller tube.

   a. True
   b. False

8. If replacement of the tube has been replaced with the same size, and then a smaller size the next step in the emergency algorithm is to remove it altogether.

   a. True
   b. False

9. If a child has had their tube removed during a tracheostomy emergency, it is unsafe to try and ventilate them using a bag, valve, mask over the stoma in the neck.
   a. True
   b. False

10. Which of the following methods may be useful when assessing for signs of breathing during a tracheostomy emergency? Select all that are correct.

   a. Look at the rise and fall of the chest
   b. Listen for breath sounds at the neck
   c. Listen for breath sounds at the mouth
   d. Fell for breath sounds at the neck
   e. Feel for breath sounds at the mouth
